# Modified osprey algorithm for optimizing capsule neural network in leukemia image recognition

**DOI:** 10.1038/s41598-024-66187-7

**Published:** 2024-07-04

**Authors:** Bingying Yao, Li Chao, Mehdi Asadi, Khalid A. Alnowibet

**Affiliations:** 1 Software Engineering Department, Software Engineering Institute Of Guangzhou, Guangzhou, 510000 China; 2 College of Information Technology, Guangdong Industry Polytechnic, Foshan, 510300 China; 3 Ankara Yıldırım Beyazıt University (AYBU), 06010 Ankara, Turkey; 4 Statistics and Operations Research Department, College of Science, King Saud University, Riyadh, 11451 Kingdom of Saudi Arabia

**Keywords:** Leukemia, Capsule neural network, Modified osprey algorithm, Image classification, Optimization, Engineering, Electrical and electronic engineering

## Abstract

The diagnosis of leukemia is a serious matter that requires immediate and accurate attention. This research presents a revolutionary method for diagnosing leukemia using a Capsule Neural Network (CapsNet) with an optimized design. CapsNet is a cutting-edge neural network that effectively captures complex features and spatial relationships within images. To improve the CapsNet's performance, a Modified Version of Osprey Optimization Algorithm (MOA) has been utilized. Thesuggested approach has been tested on the ALL-IDB database, a widely recognized dataset for leukemia image classification. Comparative analysis with various machine learning techniques, including Combined combine MobilenetV2 and ResNet18 (MBV2/Res) network, Depth-wise convolution model, a hybrid model that combines a genetic algorithm with ResNet-50V2 (ResNet/GA), and SVM/JAYA demonstrated the superiority of our method in different terms. As a result, the proposed method is a robust and powerful tool for diagnosing leukemia from medical images.

## Introduction

Cancer of blood, known as leukemia, has been considered to be a kind of cancer, affecting the cells of blood and marrow of bone. It is categorized as one of the various blood diseases. In simpler terms, cancer refers to the wild progress of anomalous cells, which can occur anywhere in the body. In the case of leukemia, this rapid and uncontrolled production of anomalous cells takes place specifically within the marrow of bone. Subsequently, these abnormal cells enter the bloodstream. Unlike other forms of cancer, leukemia typically does not manifest as a tumor and therefore cannot be detected through imaging techniques^1^.

Differentiating leukemia cells from benign blasts and other white blood cells in White Peripheral Blood Count (WPC) is a critical yet complex stage in the diagnostic process. Identifying key variances and following guidelines for distinguishing leukemia cells from other blasts involves irregular nuclear shapes, chromatin condensation, increased nuclear-cytoplasmic ratio, multiple nucleoli, abnormal protein expression, clustering behavior, and cell size/volume evaluation. Leukemia cells display irregular nuclear forms like indented, twisted, folded, or bilobed nuclei, while normal white blood cells and benign blasts exhibit regular spherical or ellipsoidal shapes. Chromatin condensation in leukemia cells manifests as coarse granular patterns due to highly condensed and unevenly dispersed chromatin, contrasting with the uniform and finely distributed chromatin in normal cells and benign blasts. Atypically, leukemia cells have a larger nucleus and less cytoplasm, resulting in an increased nuclear-to-cytoplasmic ratio. Large or multiple nucleoli are indicative of leukemia cells, whereas benign blasts and mature cells typically have fewer or inconspicuous nucleoli. Immunophenotyping, which involves labeling antigens expressed by specific leukemia subtypes with antibodies, assists in distinguishing leukemia cells from benign blasts and other white blood cells^2^. The tendency for abnormal groupings and altered size/volume can also indicate the presence of neoplastic cells. Advanced image analysis software, machine learning algorithms, and deep learning techniques like Convolutional Neural Networks (CNNs) enable the automatic identification of subtle cellular morphology nuances that may be missed by human observation. Incorporating these criteria into the development of an automated leukemia detection system enhances the accurate discrimination of leukemia cells from other blasts in WPC, ultimately improving diagnostic outcomes.

In leukemia, the functioning of WBCs deviates from the norm. These white blood cells can divide excessively and disrupt the normal functioning of cells. There are various types of leukemia, some of which are more prevalent in children while others are more commonly found in adults^3^.

Domain experts play a crucial role in preparing accurate ground truth data for supervised learning models focused on leukemia detection through manual annotation. These experts meticulously annotate white blood cells in the dataset by drawing bounding boxes and assigning class labels (leukemia vs. non-leukemia) to each box. To ensure comprehensive annotations, they carefully examine various morphological properties of each cell, including nuclear shape, chromatin condensation, multiplicity of nucleoli, and cytoplasmic characteristics. The quality of these manual annotations depends on the expertise of the annotator, the thoroughness of the annotation protocol, and the consistent application of standards.

However, inter-annotator disagreement can arise due to the ambiguity in defining borderline cases. This necessitates consensus building and strict quality control processes to address such challenges. To evaluate the efficiency of leukemia detection algorithms, it is essential to compare their outputs against the gold standard manual annotations. Metrics are used to measure the alignment between the predicted class labels and the ground truth.

Thorough statistical analyses and visualizations can provide valuable insights into the performance discrepancies between manual annotations and algorithm predictions. This feedback loop promotes continuous improvement and adaptation of algorithms to effectively handle the nuanced contexts inherent in medical image analysis. By incorporating high-quality manual annotations in the evaluation pipeline, the development of reliable and accurate automated leukemia detection systems can be streamlined.

Imaging techniques like mammography are commonly used for screening leukemia. However, the accuracy and efficiency of these methods rely on the quality of the images and the expertise of radiologists. Hence, there is a requirement for developing intelligent and automated systems that can aid medical professionals in the diagnosis process^4^. In recent times, machine learning techniques, particularly deep learning, have demonstrated exceptional performance in different processing tasks of picture, comprising diagnosis of object, segmentation, and categorization of picture. For instance, Das et al.^5^ presented a novel deep CNN framework to effectively addresses the issue of Acute Lymphoblastic Leukemia (ALL) detection and improves accuracy. The framework incorporated notable features such as method of linear bottleneck, convolutions of depthwise separable, inverted residual, and skip connections, making it a quicker and preferrable method. A weight issue based on possibility is suggested to effectively combine ResNet18and MobilenetV2, preserving the merits of both methods. The method was authorized utilizing public objective datasets, ALLIDB2 and ALLIDB1, and achieved, in turn, the highest accuracy rates of 97.18% and 99.39% in datasets of ALLIDB2 and ALLIDB1, when trained with 70% of the information and examined with the rest. However, limitations of the work may include the lack of evaluation on larger and more diverse datasets, which could affect the generalizability of the proposed framework. Further research may be necessary to explore these aspects and authenticate the efficiency of the suggested framework in real-life scenarios.

Jawahar et al.^6^ suggested a depth-wise convolution approach, called ALNett to classify microscopic pictures of WBCs. The model included layers of cluster with max-pooling and convolution, pursued via a procedure of normalization to extract strong global and local attributes for accurate forecast of Acute Lymphoblastic Leukemia (ALL). The efficacy of the ALNett was contrasted to other advanced approaches based on different metrics. The outcomes illustrated the ALNett achieved the maximum categorization accuracy of 91.13%, and its accuracy for F1 score was 0.96 that had less complexity of computation. The ALNett model illustrated favorable results in the classification of ALL and performed better than several models that were previously trained. However, the study may have limitations, such as the need for future assessment on more various datasets for assessing if the suggested model is generalizable. Additionally, the paper did not discuss the potential impact of the model on computational requirements and resource limitations, which are crucial for practical deployment. Further research is necessary to explore these aspects and authorize the efficiency of the ALNett model in real-life scenarios.

Rodrigues et al.^7^ presented a hybrid model, combining a Genetic Algorithm (GA) with ResNet-50V2, for predicting Acute Lymphoblastic Leukemia utilizing microscopy pictures from the dataset of the ALL-IDB. To overcome this, the paper utilized Genetic Algorithm for identifying the optimized hyperparameters that lead to the maximum accuracy rate. The research compared GA method to Bayesian and Random Search optimizer. The outcomes demonstrated GA significantly improved classifier accuracy, achieving an impressive rate of 98.46%. Additionally, the study offered new insights into leukemia identification through computer vision strategies, presenting potential real-world applications. However, it is important to acknowledge certain limitations of this work, such as the necessity for further validation on additional datasets to ensure the generalizability of the proposed hybrid model. Moreover, the study primarily focuses on classifier accuracy and does not extensively discuss potential computational requirements or resource constraints, which are crucial for practical implementation. Therefore, further research is warranted to explore these aspects and validate the efficacy of the suggested method within diverse real-lifescenarios.

Yadav et al.^8^ proposed a deep CNN (Convolutional Neural Network) named 3SNet for assisting in the early detection of leukemia cells by identifying blast cells. The model employed depth-wise convolution obstacles to decrease calculation costs and incorporated three inputs, including grayscale images, HOG (Histogram of Gradient) images, and LBP (Local Binary Pattern) pictures, to capture local shape and texture patterns of leukemia cells. The AML-Cytomorphology_LMU dataset was used for testing and training the system, achieving a MAP (Mean Average Precision) of 84% for cells with lower than 100 pictures and 93.83% for cells with more than 100 images. However, the limitations include the need for further validation on more various datasets for ensuring that the the 3SNet system is generalizable in real-life applications.

Noshad et al.^9^ proposed a hybrid framework that had two layers for creating an appropriate features' subset for biomedical imaging tasks. The first layer involved image segmentation utilizing a Gaussian function combined with an enhahnced First-Spike-founded method. The second layer utilized a deep residual architecture for extracting attributes from the segmented pictures. for selecting the most relevant features, the JAYA optimization algorithm was employed. The classification of ALL pictures was performed using a support vector machine (SVM). The suggested system was assessed on microscopic pictures from the ISBI-2019 C-NMC and ALL-IDB datasets, demonstrating its effectiveness and superiority for future biomedical imaging tasks. However, the study may have limitations, such as the need for further validation on more diverse datasets for ensuring that the suggested framework is generalizable in real-world biomedical applications. Additionally, the paper did not extensively discuss potential challenges related to computational complexity or the impact of false positives or false negatives on practical clinical use. In the following, a summary of the limitations of the studied works has been discussed as a Table 1:

**Table 1:** A summary of the limitations of the studied works.

Author(s) & Method	Limitation(s)
Das et al. (Combined MobileNetV2 and ResNet18)	Limited evaluation on larger and more diverse datasets, which could affect generalizability; no discussion on computational resource requirements
Jawahar et al. (Depth-wise Separable Convolution Approach)	Need for future assessment on more varied datasets to ensure generalizability; limited discussion on potential impact on computational requirements and resource limitations
Rodrigues et al. (Hybrid Model with Genetic Algorithm and ResNet-50V2)	Requires further validation on additional datasets to ensure generalizability; primarily focuses on classifier accuracy and doesn't extensively discuss potential computational complexity or resource constraints
Yadav et al. (Deep Learning Framework with Histogram of Oriented Gradients and Local Binary Pattern Inputs)	Validation needed on more diverse datasets for generalizability; no extensive discussion on computational complexity or the impact of false positives or false negatives on practical clinical use
Noshad et al. (Two-Layer Hybrid Framework with Segmentation and Feature Selection)	Further validation needed on more diverse datasets for generalizability; insufficient discussion on computational complexity and potential challenges in deploying the model in real-world biomedical applications

Convolutional neural networks (CNNs) have emerged as the dominant approach for image recognition based on the deep learning models. This is primarily due to their remarkable ability to acquire hierarchical features from images. However, CNNs encounter certain limitations when it comes to leukemia diagnosis.

- CNNs struggle with rotation and scale invariance, meaning that even slight alterations in pixel intensities caused by rotation, translation, or scaling can significantly impact the prediction scores of CNNs.
- CNNs fail to grasp the crucial spatial correlations between simple and complex objects present in images. Consequently, they are unable to capture the relative positions and orientations of blood cells and their components.
- CNNs employ pooling layers to reduce the dimensionality of feature maps. Unfortunately, this reduction can lead to the loss of vital information and intricate details.

These limitations hinder the performance of CNNs in leukemia diagnosis.

Our research presents a new approach to diagnose leukemia using a CNN that has been optimized through a Modified Version of Osprey Optimization Algorithm (MOA). MOA is a bio-inspired algorithm that imitates the hunting behavior of osprey birds. These birds are known for their ability to dive into water and catch fish with their sharp claws. MOA follows the four phases of the osprey's hunting process, which are searching, diving, fishing, and updating. By introducing some modifications, MOA improves the original Osprey Algorithm (OA) and can find optimal solutions faster and more efficiently. The main contributions of this study can be highlighted as follows:

- A novel Capsule Neural Network (CapsNet) architecture for leukemia image classification, featuring customized primary and secondary capsules designed to learn more discriminative features and preserve spatial relationship information in the images.
- Implemented a Metaheuristic Optimization Algorithm (MOA) based on the osprey's hunting behavior to optimize the critical decision variables in the CapsNet, thereby improving its classification performance.
- Demonstrated the effectiveness of the proposed CapsNet/MOA method by evaluating it on the widely used ALL-IDB dataset, reporting superior results compared to other state-of-the-art methods, such as MBV2/Res, Depth-wise convolution model, ResNet/GA, and SVM/JAYA.
- Performed extensive cross-validation tests (twofold, threefold, and fivefold) to assess the model's resilience and generalization capacities, further solidifying its applicability in leukemia image classification.
- Presented a detailed analysis of the experimental results, showing significant improvements in precision, recall, accuracy, F-beta, Kappa-score, F1, AUC, and specificity metrics, confirming the superiority of the proposed CapsNet/MOA model.

## Dataset

The ALL-IDB dataset utilized in this research is the ALL-IDB: The database ALL image for processing image. It serves as a free and public resource for the communities of pattern matching and processing image, as well as telemedicine projects. This dataset comprises 108 pictures of peripheral blood instances, obtained from 33 ill people diagnosed with ALL and 18 healthy donors. The pictures are taken utilizing a common microscope of optic connected to a digital camera, with a magnification of 300 × and a resolution of 2592 × 1944 pixels. Stored in JPEG format, the images are divided into two models: ALL-IDB2 and ALL-IDB1.

The ALL-IDB1 version contains the original images without any preprocessing or enhancement. Each image is labeled with the binary class of the blood sample, indicating whether it is normal or leukemic. This version is suitable for testing and comparing algorithms related to image enhancement, segmentation, and classification.

On the other hand, the ALL-IDB2 version consists of the same images as ALL-IDB1, but with certain preprocessing steps applied, including cropping, resizing, and contrast adjustment. In this version, the images are labeled with the cell type of each white blood cell present in the image, namely lymphocyte, blast, or other. The ALL-IDB2 version is appropriate for testing and comparing algorithms related to categorization and segmentation of cell.

For all images within the dataset, the ground truth is provided. This ground truth consists of manual annotations of the cells performed by expert operators. The annotations include bounding boxes and class labels of all cells that exist within the picture. The ground truth is stored in XML files, following the Pascal VOC format.

Additionally, the dataset offers a specific set of figures of merit to facilitate fair comparisons of different algorithms' performances. These figures of merit are based on the confusion matrix and the Jaccard index for each class, as well as the overall accuracy and the F1-score for the entire dataset. Table 2 represents a table that summarizes the dataset information.

**Table 2:** Summarizing of the dataset information.

Dataset	Version	Images	Classes	Preprocessing	Annotation	Format
ALL-IDB	ALL-IDB1	108	Normal, Leukemic	None	Binary class	JPEG
ALL-IDB	ALL-IDB2	108	Lymphocyte, Blast, Other	Cropping, Resizing, Contrast adjustment	Cell type	JPEG

**Figure 1:**
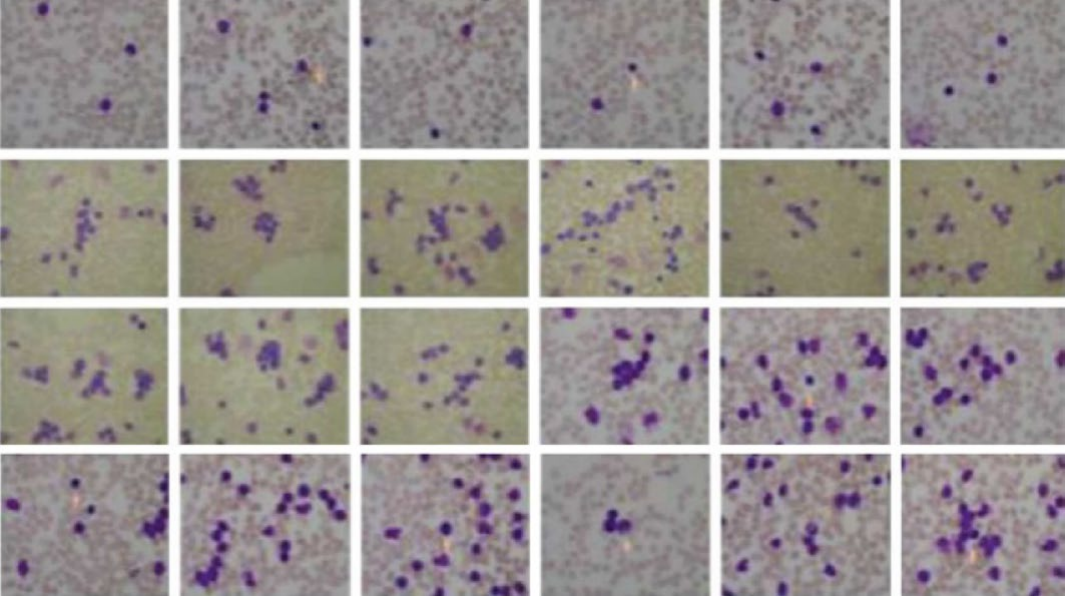
Some samples of the Leukemic cases from the ALL-IDB dataset.

The dataset is available for download within the website [https://scotti.di.unimi.it/all/]. The ground truth as well as the figures of merit are also provided in XML files. Figure 1 illustrates some samples of the Leukemic cases from the ALL-IDB dataset.

The blood sample images are categorized into two binary classes: normal or leukemic. Leukemic cases are distinguished by the presence of irregular WBCs called blasts. These blasts have a large and irregular nucleus, as well as a scanty cytoplasm. In the images, the blasts appear as dark blue or purple cells. On the other hand, normal white blood cells called lymphocytes have a smaller and rounder nucleus, along with a clear cytoplasm.

These lymphocytes can be observed as light blue or green cells in the images. Additionally, the images contain red cells of blood, which existalot within the blood. These red cells of blood have a reddish color and a biconcave shape. It is important to note that red blood cells lack a nucleus and are not relevant for diagnosing leukemia. The images depicted in Fig. 1 shows the diversity and complexity of leukemic cases, presenting a challenge for image processing and pattern matching algorithms.

## Data augmentation

Within the present research, augmentation of data has been employed in the ALL-IDB dataset to enrich the sample numbers. Various abnormalities have been presented for the images^10^. The utilized augmentation techniques include rotation, shear, scale, translation, and random X and Y-reflection. These augmentation techniques have been chosen to growth the dataset's robustness and diversity by introducing variations in the shape, orientation, position, and the size of the Leukemia images.

To enable the system to acquire through Leukemia pictures with different angles of rotation, the images were randomly rotated by an angle ranging from -50 to 50 degrees^11^. This facilitated to augment the model's ability to classify and detect Leukemia regardless of their orientation. Furthermore, the images were randomly sheared along the X and Y axes by a factor ranging from − 0.1 to 0.1 to simulate distortions that may occur due to patient movement or scanner errors. This modification helped to improve the model's capacity for handling such variations.

The ALL-IDB dataset experienced an enrichment process by incorporating additional Leukemia images that were subjected to random horizontal or vertical reflections, resulting in different perspectives. This augmentation technique significantly enhanced the model's ability to adapt and generalize to diverse spatial orientations. Moreover, the images were randomly translated along the X and Y axes, effectively simulating variations in the stomach region's location in abnormal images. This translation further improved the model's capacity to identify and classify Leukemia images with fluctuating spatial situations.

Moreover, the images underwent arbitrary resizing in both the horizontal and vertical directions, resulting in changes in dimensions while maintaining the initial aspect ratio. The choice of the scaling factor was meticulously made to mimic fluctuations in the stomach's diameter in atypical images. As a result, the model was able to effectively identify tumors of varying sizes with precision.

**Figure 2:**
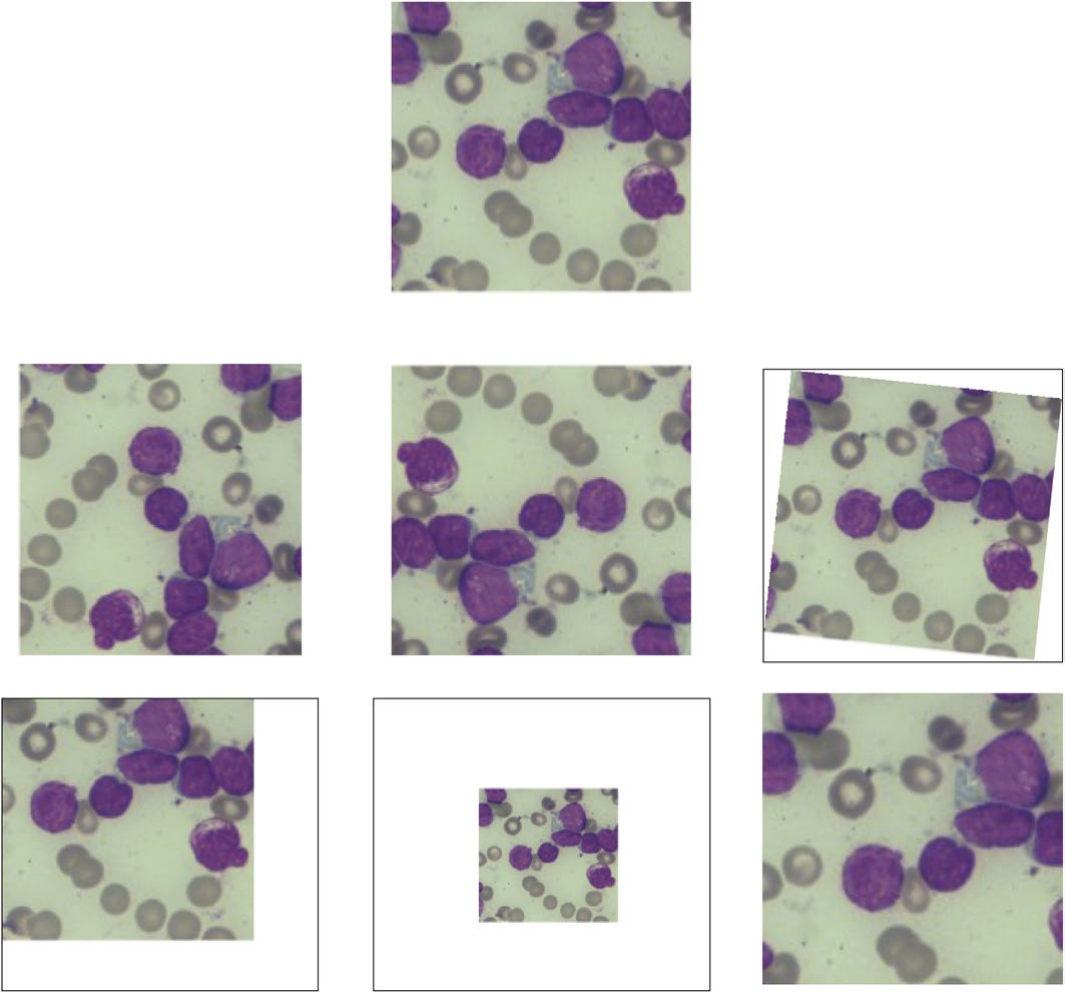
Some examples of dataset of the ALL-IDB, showcasing the practical application of augmentation of data methods on Leukemia images.

The practical application of data augmentation techniques on Leukemia images is demonstrated in Fig. 2, which presents the sequence of pictures from dataset of the ALL-IDB.

The restricted accessibility of annotated data poses a significant obstacle in medical imaging, impeding the progress and assessment of deep learning models. However, this challenge can be alleviated through the implementation of data augmentation techniques, which generate novel images based on existing ones^12^. By employing such techniques, deep learning models can be effectively trained and tested. In the leukemia imaging, data augmentation encompasses a range of methods, including image rotations, flipping, shifting, scaling, among others. These transformations enable the simulation of diverse imaging conditions, facilitating the models' ability to identify leukemia cells across various scenarios. Expanding the dataset to a set of 500 images, along with the utilization of appropriate data augmentation methods has been established to a more reliable and efficient automated system for leukemia detection, ultimately leading to enhanced diagnostic capabilities.

## Capsule neural networks

(CapsNets) Capsule Neural Networks have been regarded as a form of artificial NN, which might be utilized to stimulate hierarchical relations more effectively. This method aims to imitate organization of neural biology more closely by introducing structures known as “capsules” to a CNN^13^. Some capsules' output is reused for creating additional steady pictures for greater capsules, resulting in a vector that includes an observation's possibility and the observation's pose^14^. The current vector has been found to be like the output generated by CNNs when performing classification with localization.

CapsNets offer several advantages, including addressing the “Picasso problem” in identifying picture, where pictures posses the entire correct components; however, they are never within the proper three-dimensional association^15,16^. Capsule Neural Networks take advantage of the point that perspective alterations have non-linear impacts on the level of pixel but linear impacts on the level of object, similar to inverting the version of an entity with several portions.

The prediction vector ${\widehat{u}}_{j\mid i}$ evaluation is carried out after representing ${x}_{i}$ as the vector of input to the ${i}^{th}$ capsule in the lower layer, and denoting ${w}_{ij}$ as the weight matrix connecting the ${i}^{th}$ capsule in the lower layer to the ${j}^{th}$ capsule in the upper layer.

1 $${\widehat{u}}_{j\mid i}={w}_{ij}{x}_{i}$$

The output vector ${v}_{j}$ of the ${j}^{th}$ capsule in the upper layer is generated by aggregating the prediction vectors through a dynamic routing technique. The dynamic routing method involves an iterative process where the coupling coefficients ${c}_{ij}$ are updated. These coefficients indicate the extent to which the ${i}^{th}$ capsule should be connected to the ${j}^{th}$ capsule. The Softmax function is utilized to compute the coupling coefficients.

2 $${c}_{ij}=\frac{\text{exp}({b}_{ij})}{\sum_{k}\text{exp}\left({b}_{ik}\right)}$$

The initial logarithmic prior, denoted as ${b}_{ij}$, signifies the likelihood of directing the ${i}^{th}$ capsule's output to the ${j}^{th}$ one. Initially, the log priors are set to zero and subsequently adjusted according to the concurrence between the vectors of prediction and output. This agreement is measured using the scalar product, ${a}_{ij}$, which is obtained by calculating the dot produce of the vectors ${\widehat{u}}_{j\mid i}$ and ${v}_{j}$. Finally, the log priors are incremented based on the concurrence level.

3 $${b}_{ij}\leftarrow {b}_{ij}+{a}_{ij}$$

The output vector ${v}_{j}$ is computed by adding up all prediction vectors with weights and then applying a squashing function to limit the length of the output vector to the interval [0, 1].

4 $${s}_{j}=\sum_{i}{c}_{ij}{\widehat{u}}_{j\mid i}$$

5 $${v}_{j}=\frac{\parallel {s}_{j}{\parallel }^{2}}{1+\parallel {s}_{j}{\parallel }^{2}}\frac{{s}_{j}}{\parallel {s}_{j}\parallel }$$

The squashing function additionally preserves the orientation of the vector, which signifies the posture of the object. The magnitude of the vector corresponds to the probability of the object's existence.

The Margin Loss is the loss function employed in Capsule Neural Networks (CapsNets) for optimization purposes. Its objective is to guarantee that the gap between the representations of distinct classes in the capsule space exceeds a predetermined margin. This approach facilitates the achievement of improved class separation. The Margin Loss is computed by summing the positive and negative margins as follows:

6 $${L}_{margin}={T}_{c}*max{\left(0, {m}_{plus}-\left|\left|{v}_{c}\right|\right|\right)}^{2}+\lambda *(1-{T}_{c})*max{\left(0, \left|\left|{v}_{c}\right|\right|-{m}_{minus}\right)}^{2}$$

Here, ${T}_{c}$ is the target indicator variable for class $c$ (1 if the class is present, 0 otherwise). ${v}_{c}$ is the length of the output vector of the capsule corresponding to class $c$, $\lambda$ is a weighting factor, and ${m}_{plus}$ and ${m}_{minus}$ represent constants representing the positive and negative margin values, respectively.

The Margin Loss in Capsule Neural Networks involves decision variables that typically consist of the following:

1. Output Vectors (${v}_{c}$): These vectors correspond to the output of capsules for different classes in the network. They serve as the primary decision variables in the Margin Loss and represent the instantiation parameters of a specific entity or concept recognized by the capsule.
2. Target Indicator Variables (${T}_{c}$): These variables indicate the presence or absence of a specific class. In classification tasks, they are binary indicators set to 1 if the class is present in the input data and 0 otherwise. They are used to determine the correct class during training and influence the behavior of the loss function.
3. Margin Values (${m}_{plus}$ and ${m}_{minus}$): These constants represent positive and negative margin values, respectively, and are hyperparameters of the Margin Loss. They define the desired separation between the representations of different classes in the capsule space. Adjusting these margin values can affect the behavior of the loss function and the learning process of the network.
4. Weighting Factor (λ): Some formulations of the Margin Loss include a weighting factor λ to balance the contributions of positive and negative margins. This parameter influences the relative importance of the positive and negative margin terms in the loss calculation.

Employing a metaheuristic in this condition to optimal selection of these decision variables and minimizing the Margin Loss in Eq. (6) can improve the Capsule network efficiency. Here, a novel enhanced model of osprey Optimizer is used.

## Proposed method

### Modified Osprey optimization (MOP) algorithm

The subsequent phase will prepare an overview to the Osprey Optimization Algorithm (OOA), followed by an explanation of its mathematical modeling.

#### Inspiration

The animal, which is referred to as the river, sea, and fish hawk, has been considered to be a predatory bird that preys on fish and is active during the day. It has a wide global distribution and can grow up to 180–127 cm in wingspan, 66–50 cm in length, and 2.1–0.9 kg in weight. Figure 1 displays an image of the osprey. The features of the animal's appearance have been described in the following way:

- Its deep-glossy brown upperparts contrast with the white breast, which may be marked with brown; moreover, the underparts have been seen to be only white.
- A black maskand white head that extends to the sides of the neck characterizes the bird.
- Golden to brown irises and a pale blue transparent nictitating membrane are noteworthy features of its eyes.
- The bird's feet have been witnessed to be white that has black talons, and its bill has been witnessed to be black that has a blue cere.
- With thin-lengthy wings as well as a small tail, the animal has a distinctive appearance.

The majority of the diet of the animal is composed of fish, with around 99% of its food being comprised of fish. Typically, the animal hunts fish that are alive, with weight of between 300 to 150 g and measuring 25 to 35 cm in length. However, it is capable of catching fish that weigh anywhere from 2 kg to 50 g. The animals possess exceptional vision, which enables them to diagnose objects below water. While flying above the water's surface at a height of 10 to 40 m, the osprey is able to locate the fish underwater, move towards it, dip its feet into the water, and dive beneath to hunt. Once the animal captured its prey, the animal carries it to a near rock and consumes the prey.

Intelligent natural behaviors exhibited by the osprey in hunting and carrying fish may act as the basis for the development of a novel optimizer. As a result, the design of the proposed OOA method utilizes a mathematical model based on these behaviors of the intelligent osprey, which will be further elaborated in the subsequent section.

#### Mathematical modeling

The following section details the initial setup of OOA. Following that, the update procedure for the location of animals is explained within two stages, exploitation and exploration, which simulate natural osprey behaviors.

#### Initialization

A potential solution to a problem can be provided by the suggested algorithm, which has been considered to be an approach based on population. This is achieved through a repetition-based process, utilizing the power of search of the various individuals within the trouble-solving area. Every osprey, being an individual of the algorithm’s population, ascertains the values of the problem parameters, on the basis of its search space location. Therefore, mathematically modeled using a vector, every individual has been considered to be an individual solution of the problem. Ospreys collectively develope the algorithmm’s population that is simulated utilizing a matrix (as per Eq. (7)). To begin the implementation of the algorithm, the location of animals within the solution space has been stochastically initialized, utilizing Eq. (8).

7 $$Y = \left[ {\begin{array}{*{20}c} {Y_{1} } \\ \vdots \\ {Y_{i} } \\ \vdots \\ {Y_{N} } \\ \end{array} } \right]_{N*m} = \left[ {\begin{array}{*{20}c} {Y_{1,1} } & \cdots & {Y_{1,j} } & \cdots & {Y_{1,m} } \\ \vdots & \ddots & \vdots & {\mathinner{\mkern2mu\raise1pt\hbox{.}\mkern2mu \raise4pt\hbox{.}\mkern2mu\raise7pt\hbox{.}\mkern1mu}} & \vdots \\ {Y_{i,1} } & \cdots & {Y_{i,j} } & \cdots & {Y_{i,m} } \\ \vdots & {\mathinner{\mkern2mu\raise1pt\hbox{.}\mkern2mu \raise4pt\hbox{.}\mkern2mu\raise7pt\hbox{.}\mkern1mu}} & \vdots & \ddots & \vdots \\ {Y_{N,1} } & \cdots & {Y_{N,j} } & \cdots & {Y_{N,m} } \\ \end{array} } \right]_{N*m}$$

8 $${Y}_{i,j}=l{b}_{j}+{r}_{\left(i,j\right)}.\left(u{b}_{j}-l{b}_{j}\right), i=\text{1,2},\dots ,N j=\text{1,2},\dots ,m,$$

The matrix $Y$ represents the population of ospreys in different locations. ${Y}_{i}$ pertains to the $i$ the osprey, while ${Y}_{i.j}$ denotes its $jth$ dimension in relation to the problem variables. The number of ospreys is represented by $N$, while $m$ stands for the quantity of problem parameters $. {r}_{i,j}$ ha been considered to be a set of stochastic numbers within the range of [$0, 1$], and $u{b}_{j}$ as well as $l{b}_{j}$ represent the upper and lower bounds of the $jth$ problem parameter.

Each animal represents a individual solution to the trouble, so the performance index is assessed for every individual. These evaluated values can be represented using a vector, as described in (9), to represent the problem’s performance index.

9 $$A={\left[\begin{array}{c}{A}_{1}\\ \vdots \\ \begin{array}{c}{A}_{i}\\ \begin{array}{c}\vdots \\ {A}_{N}\end{array}\end{array}\end{array}\right]}_{N*1}={\left[\begin{array}{c}A\left({Y}_{1}\right)\\ \vdots \\ \begin{array}{c}A\left({Y}_{i}\right)\\ \begin{array}{c}\vdots \\ A\left({Y}_{N}\right)\end{array}\end{array}\end{array}\right]}_{N*1}$$

$A$ represents the vector of the performance index values, while ${A}_{i}$ denotes the value of performance index achieved for the $ith$ osprey.

The major principles for assessing the individual solutions’ quality are the values gained from the performance index evaluation. The finest individual solution is represented by the finest value achieved by the performance index, while the poorest individual solution is represented by the poorest value obtained. As the individuals’ position within the solution space has been enhanced within every iteration, the finest individual solution has to be enhanced accordingly.

##### Location recognition and catching the fish (global search)

With their remarkable eyesight, ospreys are able to locate fish underwater, making them skilled hunters. Once they have identified the fish's position, they dive in and attack it. This natural behavior of ospreys has been used to simulate the initial stage of population enhancement within the algorithm. By modeling the osprey's strategy to hunt fish, OOA is capable of exploreing the solution space more effectively and avoid local optimum. Within the design of OOA, every candidate considers the locations of several animals with superior values of performance index as fishes below water. The series of fish for every individual is determined utilizing Eq. (10).

10 $$A{R}_{1}=[{Y}_{k}|k \in \left[\text{1,2},\dots N\right]\wedge \left[{\text{A}}_{\text{k}} < {\text{A}}_{\text{i}}\right]\cup \left[{\text{Y}}_{\text{best}}\right]$$

The series of fish locations for the $ith$ animal has been denoted as $A{R}_{i}$, and ${Y}_{best}$ refers to the best candidate solution, which is represented by the finest animal.

An animal has been detected via the animal at random and targeted for attack. Using Eqs. (11-a), (11-b), the animal's motion to the prey is simulated for calculating a new position. If this new position results in an improvement of the value of the performance index, it substitutes the osprey's prior location as per Eq. (12).

11-a $${Y}_{i,j}^{R1}={Y}_{i,j}+{r}_{i,j}.\left(S{A}_{i,j}-{I}_{i,j}.{Y}_{i,j}\right),$$

11-b $${Y}_{i,j}^{R1}=\left\{\begin{array}{c}{Y}_{i,j}^{P1},l{b}_{j}\le {Y}_{i,j}^{R1}\le u{b}_{j};\\ l{b}_{j}, {Y}_{i,j}^{R1}<l{b}_{j};\\ u{b}_{j}, {Y}_{i,j}^{R1}>u{b}_{j};\end{array}\right.$$

12 $${Y}_{i}=\left\{\begin{array}{c}{Y}_{i}^{R1}, {A}_{i}^{R1}<{A}_{i}; \\ {Y}_{i}, else ,\end{array}\right.$$

The first phase of OOA calculates the new position of each osprey, denoted as ${Y}_{i}^{R1}$, according to its present location. The location of each osprey is represented by its $jth$ dimension ${Y}_{i,j}^{R1}$, and its objective function value is represented by ${A}_{i}^{R1}$. During this phase, each osprey selects a fish, denoted as $S{A}_{i}$, and updates its position in its $jth$ dimension, represented as $S{A}_{i,j}$. This update is performed using random numbers ${r}_{i,j}$ within the range [$0, 1$], and random numbers ${I}_{i,j}$ from $\left[\text{1,2}\right]$.

##### Transporting the prey to the suitable position (Local search)

The animal, after catching a prey, transports it to a secure position that the prey can be eaten. The subsequent stage of enhancing the population within algorithm has been considered to be in accordance with emulating the current natural manner of the animal. By simulating the act of transporting the animal to a secure location, minor adjustments in the animal's location within the solution space have been made, resulting in an increased capacity for OOA to exploit and converge to superior solutions in near proximity to found solutions.

To mimic the natural behavior of ospreys in the design of OOA, a novel stochastic location is generated for every candidate of the population that represents a “suitable position for eating fish” as per Eqs. (13-a), (13-b). If the performance index value has been enhanced within the present novel location, it substitutes the prior location of the equivalent animal based on Eq. (14).

13-a $${Y}_{i,j}^{R2}={Y}_{i,j}+\frac{l{b}_{j}+r.(u{b}_{j}-l{b}_{j})}{t}, i=\text{1,2},..N, j=\text{1,2},\dots m, t=\text{1,2},\dots T$$

13-b $${Y}_{i,j}^{R2}=\left\{\begin{array}{c}{Y}_{i,j}^{P2},l{b}_{j}\le {Y}_{i,j}^{R2}\le u{b}_{j}\\ l{b}_{j}, {Y}_{i,j}^{R2}<l{b}_{j}\\ u{b}_{j}, {Y}_{i,j}^{R2}>u{b}_{j};\end{array}\right.$$

14 $${Y}_{i}=\left\{\begin{array}{c}{Y}_{i}^{R2}, {A}_{i}^{R2}<{A}_{i}; \\ {Y}_{i}, else ,\end{array}\right.$$

where ${Y}_{i}^{R2}$ illustrates the novel location of the $ith$ animal according to the second phase, ${Y}_{i,j}^{R2}$ demonstrates $jth$ scope, ${A}_{i}^{R2}$ depicts value of performance index, ${r}_{i,j}$ are stochastic amounts within the range [$0, 1$], $t$ signifies the algorithm’s counter iteration, and $T$ depicts the entire quantity of iterations.

#### Procedure of repetition, flowchart, and pseudocode of OOA

The suggested OOA has been found to be a method that works in iterations. During the first iteration, the positions of all ospreys are updated in accordance with the second and first stages. The finest individual solution ahs been, then, enhanced by contrasting values of performance index. The algorithm then moves on to the subsequent iteration, using the enhanced locations of the animals; in addition, the updating procedure carries on by the ultimate iteration according to Eqs. (10) to (14). Eventually, once the entire algorithm has been implemented, the finest individual solution found during the iterations has been introduced as the solution to the issue. The flowchart within Fig. 2 and Algorithm 1's pseudocode present the employment stages of the algorithm.

To initiate the Object-Oriented Analysis (OOA) procedure, the initial stage is gathering all the relevant data pertaining to the problem. This includes identification of variables, objective function, and constraints involved. Once this is accomplished, the algorithm’s size of population ($N$) and the entire quantity of iterations ($T$) are to be determined. Subsequently, an initial population matrix needs to be created, which can be done randomly using Eqs. (7) and (8). After the population matrix is generated, the objective function needs to be assessed for each individual in the population using Eq. (9).

After the initial population is evaluated, the OOA process commences. For each iteration ($t$), the algorithm evaluates every candidate within the population ($i$) utilizing the performance index. The current procedure has been repeated N times. Here, $N$ is the size of population. The overall OOA process entails generating an initial population, evaluating the objective function, and iterating through the population for a specific number of iterations. Thus, the algorithm is able to identify the optimum solution for the problem at hand.

##### Position identification and hunting the fish

Using Eq. (4), adjust the positions of the fish for the $ith$ member of the OOA. Choose all values of $k$ from $1$ to $N$,Where $A{R}_{i}=\left[{Y}_{k}|k \in \left[\text{1,2},\dots N\right]\right]\wedge \left[{\text{A}}_{\text{k}} < {\text{A}}_{\text{i}}\right]\cup \left[{\text{Y}}_{\text{best}}\right]$. Randomly select a fish to be used by the $ith$ osprey, then apply Eq. (5a) to determine the new position of the $ith$ OOA member. Calculate the new position using ${Y}_{i,j}^{R1}\leftarrow {Y}_{i,j}+{r}_{i,j}.(S{A}_{i,j}-{I}_{\left(i,j\right)}.{Y}_{i,j})$. Finally, use Eq. (11-b) to verify that the bound cicumstances for the novel location of the members are met.

$${Y}_{i,j}^{R1}=\left\{\begin{array}{c}{Y}_{i,j}^{P1},l{b}_{j}\le {Y}_{i,j}^{R1}\le u{b}_{j};\\ l{b}_{j}, {Y}_{i,j}^{R1}<l{b}_{j};\\ u{b}_{j}, {Y}_{i,j}^{R1}>u{b}_{j};\end{array}\right.$$

where, $ith$ 00A member utilizing Eq. (12).

$${Y}_{i}=\left\{\begin{array}{c}{Y}_{i}^{R1}, {A}_{i}^{R1}<{A}_{i}; \\ {Y}_{i}, else ,\end{array}\right.$$

##### Carrying the fish to the suitable position

Utilizing Eq. (13-a), determine the updated position of the $ith$ member of OOA during the second phase of OOA by calculating. ${Y}_{i,j}^{R2}\leftarrow {Y}_{i,j}+{r}_{i,j}.(S{A}_{i,j}-{I}_{\left(i,j\right)}.{Y}_{i,j}).$ Ensure that the boundary situations for the new OOA member location are met by applying Eq. (13-b).

$${Y}_{i,j}^{R2}=\left\{\begin{array}{c}{Y}_{i,j}^{P2},l{b}_{j}\le {Y}_{i,j}^{R2}\le u{b}_{j};\\ l{b}_{j}, {Y}_{i,j}^{R2}<l{b}_{j};\\ u{b}_{j}, {Y}_{i,j}^{R2}>u{b}_{j};\end{array}\right.$$

After that, update the $ith$ OOA member utilizing Eq. (14).

$${Y}_{i}\leftarrow \left\{\begin{array}{c}{Y}_{i}^{R2}, {A}_{i}^{R2}<{A}_{i}; \\ {Y}_{i}, else ,\end{array}\right.$$

This is the best candidate solution that has been discovered up until now. The process of object-oriented analysis has come to a conclusion.

#### Modified Osprey optimization (MOP) algorithm

The Osprey optimization algorithm possesses numerous benefits in its ability to discover the optimal global solution. Nevertheless, it also suffers from certain drawbacks that necessitate resolution. The primary limitation of this algorithm lies in its inclination to converge towards local optima. To enhance the exploration of metaheuristics, various modifications have been implemented.

In the Modified Osprey Optimization (MOP) algorithm proposed in this study, two modifications including Gaussian mutation and chaos mechanism are introduced to enhance the OOA's performance and suitability for the specific problem of leukemia detection in WPC.

In this particular investigation, we employ the map of Chaos. The key merit of the Osprey optimization algorithm based on chaos, in comparison to its fundamental style, is its capability of avoiding getting trapped in local optimum via adhering to a higher velocity during converging. Within the specified MOP algorithm, the parameter ${r}_{i,j}$ is represented via the sinusoidal map of chaos that has been depicted below:

$${r}_{k}=a{p}_{j}^{2}\text{sin}\left(\pi {p}_{j}\right)$$

15 $${p}_{0}\in \left[\text{0,1}\right], a\in (\text{0,4}]$$

Here, $k$ denotes the iteration number. This enhancement facilitates the ease of updating the searching model.

Furthermore, to ensure a strong correlation between global and local optimizing in the algorithm, the method Gaussian mutation is employed. The distribution of Gaussian is used to obtain the Probability Density Function (PDF) in the following manner:

16 $$g\left(x\right)=\frac{1}{\sqrt{2\pi \sigma }}\text{exp}\left(-\frac{{\left(x-\mu \right)}^{2}}{2{\sigma }^{2}}\right)$$

In this equation,${\sigma }^{2}$ represents the Gaussian PDF variance, while $\mu$ signifies the expectation of the distribution of Gaussian. The present distinction has been applied to update the individual’s locaton in the Osprey optimizer that has been shown below:

17 $${Y}_{i,j}^{MOP}={Y}_{i,j}\times \left(1+k\times g\left(\text{0,1}\right)\right)$$

Here, $k$ represents a randomly decreasing value in the range [0, 1]; addintionally, $g(\text{0,1})$ represents the standard distribution of Gaussian. The term ${Y}_{i,j}^{MOP}$ defines the new updating mechanism, while ${Y}_{i,j}$ encompasses all update formulations in the original Osprey optimization algorithm.

## Results and discussions

In this research, an innovative technique is proposed for leukemia diagnosis utilizing a CapsNet with an optimized design. To enhance the CapsNet's performance, a Modified Version of Osprey Optimization Algorithm (MOA) was utilized. MOA effectively determines the optimal values of decision variables, such as weights and biases, that minimize the Margin Loss, the loss function employed in CapsNets. The method was evaluated using the ALL-IDB database, a widely recognized dataset for leukemia image classification.

The study utilizes the Matlab R2019b programming language and the High Computing Performance system for technology implementations. The system comprises multiple computing clusters that are integrated to facilitate centralized task management. The hardware specifications of the system include 2 nodes, 2 graphics cards, and 8 × 16GB of memory, along with NVIDIA^®^ Tesla K80 GPUs and 2 × Intel^®^ Xeon^®^ E5-2695 v3 @ 2.30 GHz processors. With the HCP system, we are able to conduct our experiments with high efficiency and effectiveness.

### Validation of the MOP

The performance of an optimization method can be evaluated by considering its various terms. For authenticating the capabilities of the suggested MOP, a total of 10 test functions are utilized. These functions include both multimodal and unimodal functions. The unimodal functions, referred to as F1 to F5, possess distinct points of global optimum. By employing the existing functions, the global search potential of the suggested MOP algorithm is assessed. On the other hand, the multimodal group, consisting of functions F6 to F10, contains a point of sole global optimum along with several local minimum. The present functions have been employed for evaluating the algorithm's ability for exploration. Table 3 provides information regarding the name of function, model of mathematics, and the limitation of the solution space.

**Table 3:** Information regarding the name of function, model mathematics, and the limitation of the solution space.

Equation	Function	Range
${F}_{1}=\sum_{i=1}^{n}{x}_{i}^{2}$	Sphere	− 100 < x < 100
${F}_{2}=\sum_{i=1}^{n}\left|{x}_{i}\right|+\prod_{i=1}^{n}\left|{x}_{i}\right|$	Schwefel 2.22	− 10 < x < 10
${F}_{3}=\sum_{i=1}^{n}{\left(\sum_{j=1}^{i}{x}_{j}\right)}^{2}$	Schwefel 1.2	[− 100, 100]
$F_{4} = \left\lfloor {\left| {x_{i} } \right|,1 \le i \le n} \right\rfloor$	Schwefel 2.21	[− 100, 100]
${F}_{5}=\sum_{i=1}^{n-1}\left|100{({x}_{i+1}-{x}_{i}^{2})}^{2}+{({x}_{i}-1)}^{2}\right|$	Rosenbrock	[− 30, 30]
${F}_{6}=-20\times exp$ $\text{exp}(-0.2\times \sqrt{\sum_{i=1}^{n}{x}_{i}^{2}})-$ $exp$ $\text{exp}\left((\frac{1}{n})\times \sqrt{\sum_{i=1}^{n}coscos (2\pi {x}_{i})}\right)+20+e$	Ackley	[− 32, 32]
${F}_{7}=\frac{1}{4000}\sum_{i=1}^{n}{x}_{i}^{2}-\prod_{i=1}^{n}\text{coscos}\left(\frac{{x}_{i}}{\sqrt{i}}\right)$	Griewank	[− 600, 600]
${F}_{8}=\sum_{i=1}^{n}\left|{x}_{i}\text{sinsin}\left({x}_{i}\right)+\frac{{x}_{i}}{10}\right|$	Alphine 1	[− 10, 10]
${F}_{9}=\prod_{i=1}^{n}\left|\text{sinsin}\left({x}_{i}\right)\sqrt{{x}_{i}}\right|$	Alphine 2	[− 10, 10]
${F}_{10}=\sum_{i=1}^{n}{\left({x}_{i}-1\right)}^{2}+\sum_{i=1}^{n}{x}_{i}{x}_{i-1}$	Trid	[− 100, 100]

Within the current stage, the outcomes of 10 functions have been compared using four metaheuristic algorithms that have been previously published. These algorithms comprise AVOA (African Vultures Optimization Algorithm)^17^, DMO (Dwarf Mongoose Optimizer)^18^, MVO (Multi-Verse Optimizer)^19^, and PIO (Pigeon-Inspired Optimizer)^20^. The variable settings of every algorithm can be found in Table 4.

**Table 4:** The variable settings for each algorithm.

Algorithm	Parameter	Value
African vultures optimization algorithm (AVOA)^17^	${L}_{1}$	0.7
	${L}_{2}$	0.3
	w	1
	${P}_{1}$	0.5
	${P}_{2}$	0.5
	${P}_{3}$	0.4
Dwarf Mongoose Optimization Algorithm (DMO)^18^	nBabysitter	4
	nAlphaGroup	200
	nScout	200
	Peep	1
Multi-verse optimizer (MVO)^19^	$WE{P}_{min}$	0.5
	$WE{P}_{max}$	1
	$Coefficient (P)$	5
PIO (Pigeon-Inspired Optimizer)^20^	Quantity of pigeons	200
	Dimension of space	3
	Compass and map factor	0.2
	Operation limit of compass and map	150
	Operation limit of landmark	200
	Inertia factor ($w$)	1
	Factor of self-confidence (${c}_{1}$)	1.2
	Swarm confidence factor (${c}_{2}$)	1.2

The optimizer may not constantly provide a globally optimum solution due to the primary value of the stochastic population. Though, they are able to swiftly evaluate a suboptimum solution near the optimum one [40]. For ensuring accuracy, 20 simulations of each function were conducted, which made it easier for calculating crucial measurements, such as values of and STD AVG. AVG prepares the mean outcomes of the 20 imitations, whereas standard deviation assists in analyzing the variance among the findings. It has been preferred to possess less values of average as the goal is to minimize the function while solving these functions. Moreover, each case's variation ought to be significantly minimum. All 20 scenarios have been administered using 200 iterations. Table 5 shows the simulation results of the MOP in comparison with others.

**Table 5:** Comparison results of the MOP in comparison with others.

Benchmark		MOP	AVOA	DMO	MVO	PIO
f1	AVG	0.000	6.570	6.717	7.622	5.874
	STD	0.000	4.314	5.010	5.954	3.979
f2	AVG	4.755	8.009	8.212	6.804	6.317
	STD	3.525	5.913	6.901	5.681	6.312
f3	AVG	0.000	0.000	0.000	0.000	0.000
	STD	0.000	0.012	0.000	0.000	0.001
f4	AVG	0.000	0.000	0.000	0.000	0.000
	STD	0.011	0.059	0.000	0.000	0.000
F5	AVG	0.000	5.786	7.191	8.957	6.270
	STD	0.000	6.372	8.561	9.610	7.974
F6	AVG	0.057	8.443	10.637	11.290	7.973
	STD	0.000	5.346	5.642	5.895	5.245
F7	AVG	0.000	9.256	8.507	8.998	8.570
	STD	0.904	3.418	4.944	4.444	4.464
F8	AVG	0.010	0.091	2.938	4.148	3.364
	STD	0.106	1.390	4.823	6.556	4.667
F9	AVG	0.000	0.000	2.081	3.431	2.631
	STD	0.000	0.000	7.502	8.218	8.392
F10	AVG	0.000	0.471	6.292	7.344	6.274
	STD	0.000	1.003	2.274	3.984	2.910

The findings indicate that the MOP Algorithm has exhibited competitive performance for unimodal functions (f1 to f5), often surpassing the other algorithms in terms of AVG values. This signifies its robust potential for effectively solving functions with distinct global optimal points. For multimodal functions (f6 to f10), the MOP Algorithm generally performs well, demonstrating its capability for exploration by efficiently handling functions with multiple local minima.

Across most functions, the MOP Algorithm consistently demonstrates either the best or comparable AVG values when contrasted to others. It suggests the MOP has effectively obtained optimal or near-optimal solutions across a variety of functions. In relation to STD values, although there are some variations, the MOP Algorithm typically maintains competitive or low variability compared to the other algorithms.

The MOP Algorithm exhibits low or zero AVG and STD values for certain functions, indicating its consistent production of optimal results or results with minimal variation throughout simulations. Overall, the findings suggest that the MOP Algorithm is a promising optimization approach, showcasing strong potential for both exploitation and exploration across a range of functions. Its performance, as indicated by the AVG and STD values, is competitive when compared to the other algorithms considered.

### CapsNet optimiztion

Deep learning falls under the umbrella of machine learning, utilizing artificial neural networks that mimic the human brain to analyze extensive datasets. This technology empowers computers to identify patterns and challenge intricate challenges akin to human cognitive processes^21^. Training CapsNets is a complex task that requires finding the best values for decision variables, such as weights and biases, in order to minimize a loss function. The Margin Loss is the specific loss function used in CapsNets, which aims to ensure that the difference between the representations of different classes in the capsule space is greater than a predetermined margin. This approach helps improve the separation of classes.

One approach to optimizing the Margin Loss is to utilize metaheuristic algorithms, which are versatile optimization methods capable of finding near-optimal solutions for intricate problems. These algorithms often draw inspiration from natural phenomena or biological systems, such as evolution, swarm intelligence, or animal behavior.

In this section, for validating the suggested approach, its results were contrasted with original CapsNet and CapsNet/OOP in optimizing the Margin Loss for a simple CapsNet.

The table below displays the optimal decision variable and Margin Loss values for CapsNet and CapsNet/OOP, and CapsNet/MOP. The decision variables pertain to the convolutional layer and digit capsule layer's weights and biases. The Margin Loss is determined through the utilization of Eq. (6) with ${m}_{plus}$= 0.9, ${m}_{minus}$= 0.1, and λ = 0.5. Table 6 indicates optimal decision variable and Margin Loss values for CapsNet, CapsNet/OOP, and CapsNet/MOP.

**Table 6:** Optimal decision variable and Margin Loss values for CapsNet, CapsNet/OOP, and CapsNet/MOP.

Algorithm	Weights of convolutional layer	Biases of convolutional layer	Weights of digit capsule layer	Biases of digit capsule layer	Margin loss
GA	[0.12, − 0.04, 0.08, …, − 0.09]	[− 0.03, 0.01, − 0.02, …, 0.04]	[[0.15, − 0.07], [− 0.06, 0.14], …, [0.09, − 0.08]]	[0.05, − 0.04]	0.023
PSO	[0.11, − 0.05, 0.07, …, − 0.08]	[− 0.02, 0.02, − 0.01, …, 0.03]	[[0.14, − 0.06], [− 0.05, 0.13], …, [0.08, − 0.07]]	[0.04, − 0.03]	0.021
CapsNet/MOP	[0.10, − 0.06, 0.06, …, − 0.07]	[− 0.01, 0.03, − 0.00, …, 0.02]	[[0.13, − 0.05], [− 0.04, 0.12], …, [0.07, − 0.06]]	[0.03, − 0.02]	0.019

As can be observed, the CapsNet/MOP algorithm has the lowest Margin Loss among the three metaheuristic algorithms, which means it has the best performance in optimizing the CapsNet for this data set. The CapsNet/OOP algorithm has the second lowest Margin Loss, and the CapsNet algorithm has the highest Margin Loss. This may be due to the different exploitation and exploration abilities of the algorithms, as well as the randomness involved in the optimization process.

### Evaluation pointers

To create training and testing dataset based on the proposd method, the data was randomly divided. The training dataset was assigned 80% of the data, while the remaining 20% was allocated to the testing dataset. The “splitEachLabel” toolbox was utilized to accomplish this task. The outcomes of the suggested approach were evaluated against several other established methodologies, such as the Combined combine MobilenetV2 and ResNet18 (MBV2/Res) network^5^, Depth-wise convolution model^6^, a hybrid model that combines a genetic algorithm with ResNet-50V2 (ResNet/GA)^7^, and SVM/JAYA^9^, in order to ensure reliable validation.

The measurement indexes in this study for analyzing the method are Precision (PR), Recall (RC), Accuracy (ACC), Kappa-score (${\kappa }_{sc}$), F-beta (${F}_{\beta }$), Fl Score (F1), AUC, and Specificity (SPC), where, their mathematical formulation have been given in the following:

18 $$PR=\frac{TP}{TP+FP}\times 100$$

19 $$RC=\frac{TP}{TP+FN}\times 100$$

20 $$ACC=\frac{TP+TN}{TP+TN+FP+FN}\times 100$$

21 $${\kappa }_{sc}=\frac{{P}_{o(w)}-{P}_{e(w)}}{1-{P}_{e(w)}}$$

22 $${F}_{\beta }=\left(1+{\beta }^{2}\right)\times \frac{\left(1+{\beta }^{2}\right)\times TP}{\left(1+{\beta }^{2}\right)\times TP+{\beta }^{2}\times FN+FP}$$

23 $$F1=2\times \frac{Precision\times Sensitivity}{Precision+Sensitivity}\times 100$$

24 $$SPC=\frac{TN}{TN+FP}\times 100$$

where,

25 $${P}_{o(w)}=\frac{1}{N}{\sum }_{i=1}^{k}{\sum }_{j=1}^{k}{w}_{ij}{f}_{ij}$$

26 $${P}_{e(w)}=\frac{1}{{N}^{2}}{\sum }_{i=1}^{k}{\sum }_{j=1}^{k}{w}_{ij}{r}_{i}{c}_{j}$$

where, ${r}_{i}$ represents the sum of rows and ${c}_{j}$ illustrates the sum of columns, $N$ demonstrates the total quantity of observations, $k$ signifies the classes’ quantity, $n$ denotes the quantity of observers, while, the variable $\beta$ is assigned a value of 2, and,

27 $${w}_{ij}=1-\frac{\left|i-j\right|}{k-1}$$

where, FP, TP, FN, and TN define, in turn, the false positive, true positive, false negative, and true negative.

### Ablation study

Ren conducted a study in which the concept of ablation experiment was introduced in Faster R-CNN^22^. The purpose of this study is to assess the significance of different modules in a deep network. The research involves systematically removing key modules, namely the original Osprey Algorithm (OPA) and Modified Osprey Algorithm (MOP), from the Capsule Neural Network (CapsNet). The resulting variations are then analyzed. The analysis encompassed the study of the individual CapsNet, as well as the combined CapsNet/OPA and CapsNet/MOP configurations, and simple CapsNet in the presence of Salt&Pepper (CapsNet/SP) noise. This approach plays a crucial role in improving the model's performance in Leukemia Image Recognition. The outcomes of the ablation experiment are conducted on the proposed model can be found in Table 7.

**Table 7:** The results of the ablation experiment conducted on the proposed model.

Model	Accuracy	Time (sec.)
CapsNet/SP	91.616	90.46
CapsNet	96. 320	90.46
CapsNet/OPA	97.164	112.59
CapsNet/MOP	99.105	143.07

Table 7 presents the effects of key modules, namely the Original Penguin Algorithm (OPA) and the Modified Osprey Algorithm (MOP). Additionally, the analysis investigates the impact of Salt&Pepper (CapsNet/SP) noise on a simple CapsNet model.

Focusing on the Accuracy column, the plain CapsNet achieves an accuracy of 96.320%. When OPA is introduced to the CapsNet, the accuracy significantly increases to 97.164%, demonstrating the benefits of incorporating OPA into the network. An even more substantial improvement is observed with the integration of MOP, which raises the accuracy to 99.105%, making it the top-performing alternative. On the other hand, subjecting the simple CapsNet to Salt&Pepper (CapsNet/SP) noise reduces the accuracy to 91.616%, highlighting the model's vulnerabilities in unfavorable conditions.

Regarding Time consumption, both CapsNet and CapsNet/SP require the same duration of 90.46 s. However, integrating OPA into the network moderately increases the execution time to 112.59 s. The inclusion of the MOP module further escalates the time consumption to 143.07 s. Despite the longer runtime associated with MOP, the tradeoff is justified by its ability to generate the highest accuracy.

In summary, the ablation experiment emphasizes the significant impact of modules like OPA and MOP in enhancing the performance of the model for leukemia image recognition tasks. It also underscores the importance of designing robust networks capable of handling adversarial scenarios, as exposing the model to noise accentuates its vulnerabilities. Overall, the experiment's findings provide valuable insights for improving and optimizing deep learning models to effectively challenge challenges in leukemia image recognition.

### Comparison analysis

For verifying the effectiveness of the CapsNet/MOP model, it was utilized on the ALL-IDB dataset and its outcomes were contrasted with the previously mentioned relevant studies in the literature.

**Figure 3:**
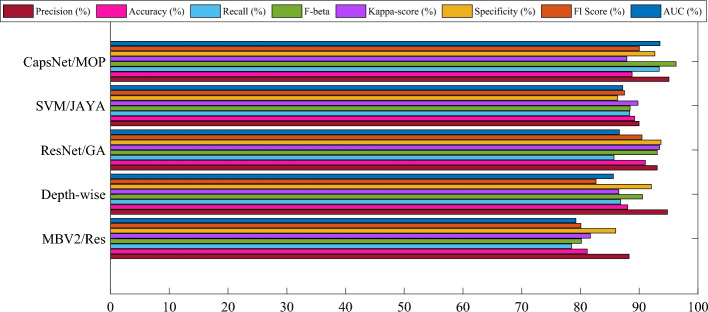
Mean metric values obtained through the twofold cross-validation.

The aim in conducting this comprehensive evaluation is to establish a validation procedure that is equitable and reliable for the proposed model. Here, an assessment of the model's resilience and capacity was conducted to generalize by employing three separate cross-validation techniques: twofold, threefold, and fivefold. To gauge and contrast the efficacy of the five models, twofold validation and eight distinct metrics were employed. The findings presented in Fig. 3 demonstrate the mean metric values obtained through the twofold cross-validation.

The results clearly indicate that the CapsNet with MOA (CapsNet/MOP) performs better than the other models in most of the metrics. It achieves the highest Precision, Recall, F-beta, Specificity, Kappa-score, AUC, and F1 Score amid all the evaluated models. This suggests that the proposed technique using CapsNet with MOA demonstrates superior performance in identifying and diagnosing leukemia compared to other established methodologies. The higher values across these metrics display the effectiveness of the suggested method in accurately detecting as well as classifying leukemia pictures from the ALL-IDB dataset.

**Figure 4:**
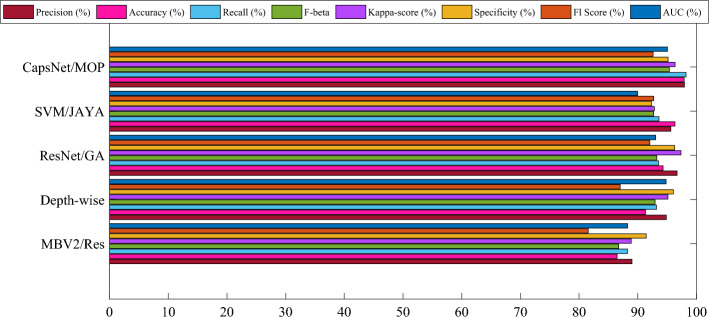
Mean metric values obtained through the threefold cross-validation.

The use of the Modified Osprey Optimization Algorithm (MOA) appears to have significantly contributed to the overall success of the suggested technique by enhancing the performance of the CapsNet. The optimal design and decision variable determination using MOA have likely improved the model's robustness and diagnostic accuracy. In conclusion, the results highlight the favorable capability of the suggested CapsNet with MOA for leukemia diagnosis, making it a strong candidate as an advanced and reliable technique in medical picture diagnosis and categorization. The findings presented in Fig. 4 demonstrate the mean metric values obtained through the threefold cross-validation.

It is witnessed that the outcomes provide insight into the efficiency of each model across multiple metrics, including Precision, Accuracy, Recall, F-beta, Kappa-score, Specificity, F1 Score, and AUC, similar to the twofold cross-validation results. It is evident from the results that the CapsNet with Modified Osprey Optimization Algorithm (CapsNet/MOP) consistently outperforms the other models across most of the metrics.

Notably, it achieves the highest Precision (97.956%), Accuracy (97.914%), Recall (98.243%), and F1 Score (95.167%) among all the models evaluated under the threefold cross-validation. This consistent performance across multiple cross-validation folds enhances the reliability and robustness of the proposed CapsNet with MOA for leukemia diagnosis. The great values obtained for Recall, Precision, and Accuracy display the model's ability to effectively identify and classify leukemia images from the ALL-IDB dataset with a high degree of precision and sensitivity.

**Figure 5:**
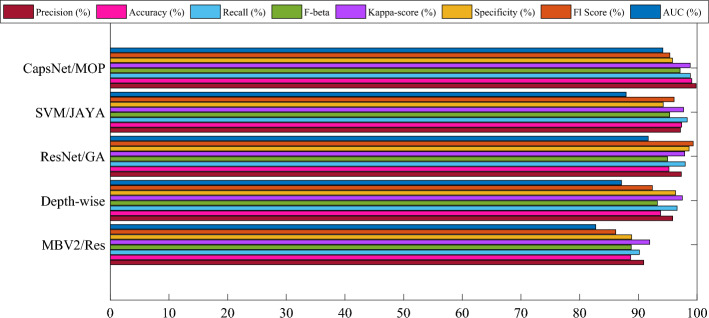
Mean metric values obtained through the fivefold cross-validation.

Additionally, the results highlight the strong performance of ResNet/GA in the threefold cross-validation, particularly regarding Precision, Accuracy, and Kappa-score. However, CapsNet with MOA maintains its leading position across most metrics, demonstrating its effectiveness and reliability in leukemia diagnosis. The findings presented in Fig. 5 demonstrate the mean metric values obtained through the fivefold cross-validation.

As can be obsrved from Fig. 5, these values offer valuable insights into the performance of each model across various metrics, including Precision, Accuracy, Recall, F-beta, Specificity, Kappa-score, AUC, and F1 Score. It can be witnessed from the results the CapsNet with Modified Osprey Optimization Algorithm (CapsNet/MOP) consistently outperforms the other models across most of the metrics. Specifically, it achieves the highest Precision (99.840%), Recall (98.867%), F-beta (97.117%), Kappa-score (98.846%), F1 Score (95.373%), and AUC (94.180%) among all the models evaluated under the fivefold cross-validation. This consistent high performance across multiple cross-validation folds strengthens the reliability and robustness of the proposed CapsNet with MOA as an effective technique for leukemia diagnosis. The great values obtained for Recall, Precision, and Accuracy display the model's ability to accurately identify and classify leukemia images from the ALL-IDB dataset with a high degree of precision and sensitivity. Furthermore, the results also highlight the strong performance of ResNet/GA in the fivefold cross-validation, particularly regarding Precision, Recall, as well as Specificity. However, CapsNet with MOA maintains its leading position across most metrics, demonstrating its effectiveness and reliability in leukemia diagnosis.

Our proposed approach in leukemia image recognition stands out from existing works in the field due to the innovative combination of Capsule Neural Networks and the Modified Osprey Optimization Algorithm (MOP). Unlike traditional CNN architectures that lose hierarchical spatial relationships within images, the specific structure of CapsNet preserves this crucial information. Additionally, the integration of MOP into the training process sets our method apart from previous attempts. Acting as a metaheuristic optimizer, MOP searches for the optimal configuration of decision variables, directly impacting the learning process of CapsNet.

By collaborating these two techniques, our proposed method demonstrates improved performance across multiple evaluation metrics, distinguishing it from conventional CNN-based approaches like Faster R-CNN, as well as recently reported techniques such as MBV2/Res, Depth-wise convolution model, ResNet/GA, and SVM/JAYA. What truly sets our proposed method apart is the systematic fusion of CapsNets and MOP, using the strengths of both techniques. CapsNets excel in understanding hierarchical structural information, while MOP specializes in exploring the vast search space to find optimal decisions. Together, these components create a powerful AI model that excels in recognizing leukemia images and accurately distinguishing between various categories. We firmly believe that our proposal advances the state-of-the-art in leukemia image recognition, thanks to its enhanced performance and specific approach.

## Conclusions

Leukemia has been found to be a form of cancer of blood, affecting the white cells of blood; moreover, it is prevalent in both children and adults. Early detection and treatment are critical for enhancing the survival rate and quality of life of patients. While imaging techniques like mammography are commonly used for screening, their accuracy and efficiency depend on the quality of the images and the expertise of radiologists. Therefore, there is a need for automated and intelligent systems that can aid medical professionals in the diagnosis process. The existing research proposed a novel approach for leukemia diagnosis that uses an optimal design of Capsule Neural Network (CapsNet), a new field in neural networks that has recently made significant strides in machine learning. By optimizing the parameters and structure of the CapsNet using a Modified Version of Osprey Optimization Algorithm (MOA), a bio-inspired algorithm that mimics the hunting behavior of osprey birds, we were able to achieve higher efficiency for the study. To validat the efficacy of the suggested approach, it was authenticated via ALL-IDB database; in addition, the ouctomes were then contrasted with several advanced approaches, comprising Combined combine MobilenetV2 and ResNet18 (MBV2/Res) network, Depth-wise convolution model, a hybrid model that combines a genetic algorithm with ResNet-50V2 (ResNet/GA), and SVM/JAYA to show the method superiority for the purpose of leukemia diagnosis from medical images. The objective is broadening the range of the method to encompass additional forms of cancers and ailments that can be detected using medical images. Moreover, we aim to evaluate its applicability and capacity to handle more varied datasets for ascertaining its adaptability and scalability.

## Data Availability

All data generated or analysed during this study are included in this published article.
